# Recent progress in advanced optical materials based on gadolinium aluminate garnet (Gd_3_Al_5_O_12_)

**DOI:** 10.1088/1468-6996/16/1/014902

**Published:** 2015-02-18

**Authors:** Ji-Guang Li, Yoshio Sakka

**Affiliations:** Advanced Materials Processing Unit, National Institute for Materials Science, 1-2-1 Sengen, Tsukuba, Ibaraki 305-0047, Japan

**Keywords:** gadolinium aluminate garnet, lattice stabilization, down-/up-conversion phosphors, single crystal, transparent ceramic, scintillator, energy transfer

## Abstract

This review article summarizes the recent achievements in stabilization of the metastable lattice of gadolinium aluminate garnet (Gd_3_Al_5_O_12_, GAG) and the related developments of advanced optical materials, including down-conversion phosphors, up-conversion phosphors, transparent ceramics, and single crystals. Whenever possible, the materials are compared with their better known YAG and LuAG counterparts to demonstrate the merits of the GAG host. It is shown that novel emission features and significantly improved luminescence can be attained for a number of phosphor systems with the more covalent GAG lattice and the efficient energy transfer from Gd^3+^ to the activator. Ce^3+^ doped GAG-based single crystals and transparent ceramics are also shown to simultaneously possess the advantages of high theoretical density, fast scintillation decay, and high light yields, and hold great potential as scintillators for a wide range of applications. The unresolved issues are also pointed out.

## 1. Introduction

**Figure 1.**
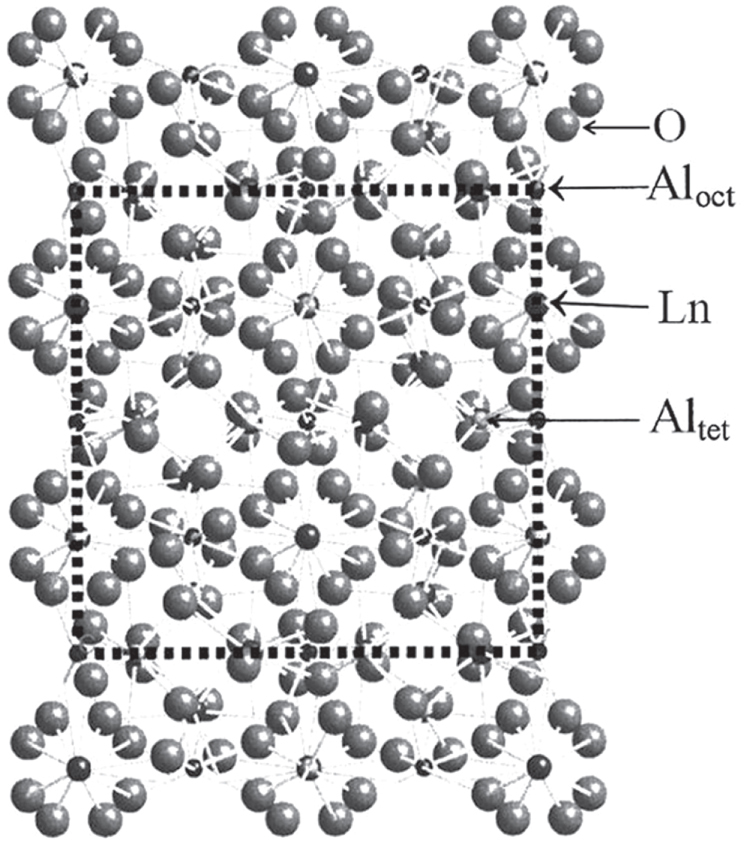
A schematic illustration of the crystal structure of LnAG, where Al_oct_ and Al_tet_ represent the Al atoms taking octahedral and tetrahedral lattice sites, respectively. Adapted with permission from [1], copyright 1999 by the American Physical Society.

Rare-earth aluminate garnets, having a general formula of Ln_3_Al_5_O_12_ (LnAG, Ln: lanthanide and Y), are an important family of multi-functional ceramic materials. The compounds crystallize in a bcc structure (space group: 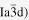 with 160 (80) atoms in the cubic (primitive) cell, where the Ln occupies the 24c sites (D_2_ point symmetry, CN = 8; CN: coordination number) and the oxygen atoms take the 96h sites. The Al atoms have two positions to reside on: the 16a sites with an octahedral point symmetry (C_3*i*_, 40%; CN = 6) and the 24d sites with a tetragonal point symmetry (S_4_, 60%; CN = 4) [1]. The garnet structure can be viewed as a framework built up via corner sharing of the Al–O polyhedra, with the Ln residing in dodecahedral interstices [1]. A schematic diagram of the crystal structure is shown in figure 1.

YAG might be the best-known garnet compound owing to its excellent chemical stability, high creep resistance, optical isotropy, and particularly the ability to accept substantial trivalent Ln^3+^ for diverse optical functionalities. In the bulk form, the YAG:Nd single crystal is one of the most widely used solid laser materials since its discovery in the 1960s [2]. Transparent YAG:Nd ceramics that are equal to or superior to single crystals in optical transmittance and laser performance have also been successfully developed since the 1990s via advanced powder processing and sintering technologies [3, 4]. YAG:Ho^3+^ and YAG:Er^3+^ are important infrared (IR) laser materials for medical surgery, since their fluorescence lines (∼2 *μ*m for Ho^3+^ and ∼3 *μ*m for Er^3+^) match well with the water absorptions of the human body [5]. Transparent YAG:Ce^3+^ ceramic is nowadays being developed as an encapsulant for white-light emitting diodes (LEDs) to replace the widely used but readily degradable resin sealant [6]. Since the parity-law allowed 4f^0^5d^1^ → 4f^1^5d^0^ transition of Ce^3+^ has a very short fluorescence lifetime of ∼10–100 ns and the intrinsic quenching temperature of Ce^3+^ in YAG is very high (>700 K) [7], YAG:Ce^3+^ transparent ceramic has been considered as a scintillator [8], but does not seem to have a satisfactory stopping power for the incident radiations (x-, *α*- or *γ*-rays) owing to the relatively low theoretical density of YAG (∼4.55 g cm^−3^) and the small atomic weight of Y (∼89). In the powder form, rare-earth (Eu^3+^, Tb^3+^, Ce^3+^ etc) activated YAG is being widely studied and used as phosphors for fluorescent lamps, field emission displays (FEDs), and white LEDs.

The occurrence and thermal stability of compounds in the Ln_2_O_3_–Al_2_O_3_ binary system heavily depend on the ionic radius of Ln^3+^, conforming to lanthanide contraction. Earlier studies by Mizuno *et al* [9–13] on the phase diagram of Ln_2_O_3_–Al_2_O_3_ found the two intermediate compounds of Ln_2_O_3_·11Al_2_O_3_ (*β*-Al_2_O_3_ type) and LnAlO_3_ (commonly referred to as aluminate perovskite or LnAP, an orthorhombic modification of ideal perovskite) for the large ions of La^3+^–Nd^3+^, the two compounds of LnAP and monoclinic Ln_4_Al_2_O_9_ (commonly called LnAM) for the intermediately sized Sm^3+^–Gd^3+^, and the three compounds of LnAM, LnAP, and LnAG for the small Ln^3+^ ions of Tb^3+^–Er^3+^. Though GdAG (further abridged as gadolinium aluminate garnet (GAG) hereafter) was not identified in the work of Mizuno *et al*, it was successfully synthesized via flux growth by Van Uitert *et al* [14] and Manabe and Egashira [15] for potential optical applications. Later on, Shishido *et al* [16] found via annealing Gd_2_O_3_·5/3Al_2_O_3_ amorphous glass that GAG is metastable and would completely decompose to *α*-Al_2_O_3_ and GdAP (Gd_3_Al_5_O_12_ → Al_2_O_3_ + 3GdAlO_3_) upon prolonged annealing at 1500 °C. A recent work by Li *et al* [17] further showed that the stoichiometric GAG synthesized via low temperature combustion starts to decompose at ∼1300 °C. All these studies point to the fact that thermodynamically stable LnAG only exists for the Ln^3+^smaller than Gd^3+^ and Gd^3+^ is the boundary for a LnAG to be formed. This is understandable from the crystal structure shown in figure 1. That is, the dodecahedral interstice has a certain geometric shape and dimension, and thus a size limit exists for Ln^3+^ to enter the space without disintegrating the Al–O framework. Mainly due to its structural metastability, GAG has been much less explored than YAG for its properties and applications, though its specific heat and thermal expansion coefficient were experimentally determined by Chaudhury *et al* [18]. Compared with YAG, however, GAG may hold a number of merits for optical applications: (1) the intrinsic ^8^S_7/2_ → ^6^I_*J*_ transition of Gd^3+^ (usually centered at ∼275 nm) can be utilized as a new excitation source for some types of rare-earth activators, and enhanced luminescence may also be attained via an efficient energy transfer from Gd^3+^ to the activator [19–22], (2) the GAG lattice is more covalent than YAG due to the lower electronegativity of Gd^3+^ (*χ* = 1.20) than Y^3+^ (*χ* = 1.22), which may produce new emission features and result in improved emission intensity, and (3) GAG has a significantly higher theoretical density (5.97 g cm^−3^) than YAG (4.55 g cm^−3^) and the atomic weight of Gd (157, close to the 175 of Lu) is much higher than Y, and thus GAG is more desirable for scintillation applications. Similar to the growth of single crystals and sintering of transparent ceramics, a reasonably high processing temperature is usually needed to produce high quality phosphors through crystal perfection. In this context, lattice stabilization becomes a prerequisite for any practical application of GAG in advanced optical materials. This review article summarizes the recent achievements in GAG, including lattice stabilization via doping and its application in down-/up-conversion (UC) phosphors and transparent ceramic/single crystal scintillators.

## 2. Lattice stabilization of GAG by modifying the Gd/Al sites

**Figure 2.**
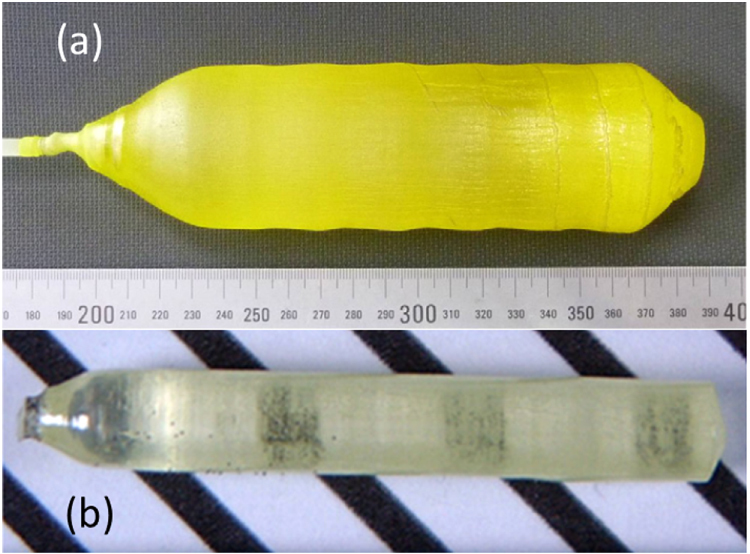
Appearance of the Gd_3_(Al_2_Ga_3_)O_12_ single crystals doped with 1 at% of Ce^3+^ (a) and 1 at% of Pr^3+^ (b). Part (a) reproduced with permission from [24] and part (b) reproduced with permission from [25], copyright 2012 by Elsevier.

There are two primary ways to stabilize the garnet lattice of GAG, as can be perceived from the crystal structure shown in figure 1, with the first one partially replacing the Al sites with suitably larger trivalent ions to enlarge the dodecahedral interstices via forming Gd_3_(Al_1−*x*_M_*x*_)_5_O_12_ solid solution and the second one being partially replacing Gd^3+^ with a smaller Ln^3+^ to form (Gd_1−*x*_Ln_*x*_)_3_Al_5_O_12_. Ga^3+^ is the main choice in the former case, and Gd_3_Ga_5_O_12_ (GGG), known as a thermodynamically stable garnet host for phosphors and solid lasers [2], is an extreme example. The effectiveness of Ga^3+^ doping was experimentally demonstrated by Chiang *et al* [23], who found that phase-pure garnet can be crystallized from chemically precipitated precursors at ∼1400 °C in the presence of 10 at% of Ga^3+^ and the crystallization temperature decreases to 1300 °C with 20 at% of Ga^3+^ addition. Without Ga^3+^ doping, only a phase mixture of LnAP, LnAG and amorphous alumina was formed. By applying the same stabilization strategy, Kamada *et al* were able to grow two-inch-diameter Gd_3_(Al_2_Ga_3_)O_12_:Ce^3+^ single crystals by the Czochralski (Cz) method using [100] oriented seeds [24] and Gd_3_(Ga,Al)_5_O_12_:Pr^3+^ single crystals by a micro-pulling down (*μ*-PD) technique [25] (figure 2). Though Ga^3+^ was thought to exclusively replace Al^3+^ in these studies, atomistic modeling using the static lattice computational approach and pairwise (Buckingham) interatomic potentials by Maglia *et al* [26] revealed that Ga^3+^, though it prefers to take the octahedral Al^3+^ site, can also be inserted into the dodecahedral position of Gd^3+^ with the generation of anti-site defects owing to its relatively large ionic radius. In addition, suppressing activator oxidation (such as Pr^3+^, Ce^3+^, and Tb^3+^) and Ga^3+^ reduction should be made at the same time to avoid lattice defects and deterioration of optical performance.

**Figure 3.**
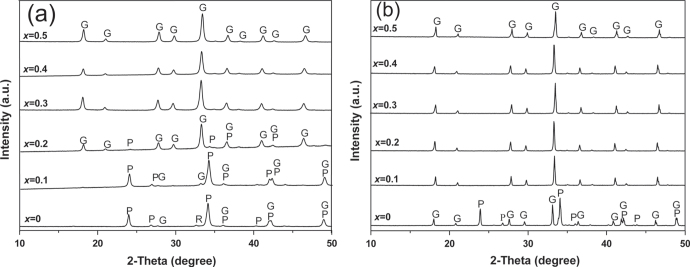
The effects of Lu content (*x* value) on phase evolution of (Gd_1−*x*_Lu_*x*_)AG solid solution. Parts (a) and (b) are for calcination temperatures of 1000 and 1500 °C, respectively. Reproduced with permission from [17], copyright 2012 by the American Ceramic Society.

Lu^3+^ (0.0977 nm for CN = 8) is the tiniest Ln^3+^ ion and would thus be the most effective to stabilize GAG via replacing the Gd^3+^ site to form a (Gd_1−*x*_Lu_*x*_)AG solid solution. With coprecipitated carbonate precursors, Li *et al* [17] thoroughly studied the effects of Lu content on phase evolution and also properties of the resultant (Gd_1−*x*_Lu_*x*_)AG (*x* = 0–0.5). It was shown that the garnet phase generally crystallizes via LnAM and LnAP intermediates, as is commonly observed for YAG, but the crystallization temperature substantially decreases towards a higher Lu content. With *x* = 0.3–0.5, phase-pure garnet can even be crystallized at a temperature as low as 1000 °C (figure 3(a)), revealing the significant effectiveness of Lu^3+^ doping. Again, only a phase mixture of LnAG, LnAP and amorphous Al_2_O_3_ was produced in the absence of Lu^3+^ (figure 3(b)).

**Figure 4.**
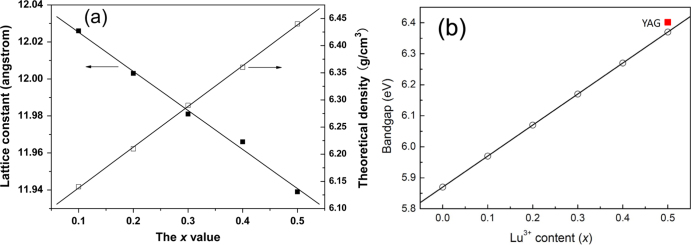
Lattice constant and theoretical density (a) and bandgap (b) of the (Gd_1−*x*_Lu_*x*_)AG solid solution, as a function of the Lu content. Part (a) reproduced with permission from [17], copyright 2012 by the American Ceramic Society.

A simultaneous advantage of Lu doping is that it improves the already high theoretical density of GAG (5.97 g cm^−3^). The (Gd_0.5_Lu_0.5_)AG solid solution, for example, reaches the high value of ∼6.44 g cm^−3^ (figure 4(a)), being close to that of the heavy LuAG (6.73 g cm^−3^). Since Gd is commercially much cheaper than Lu, the (Gd_1−*x*_Lu_*x*_)AG solid solutions may replace LuAG to be used as cost effective and high density scintillation materials. Assayed from UV/vis absorption, the (Gd_1−*x*_Lu_*x*_)AG solid solutions were found to have increasing optical bandgaps of ∼5.87, 5.97, 6.07, 6.17, 6.27, and 6.37 eV with increasing *x* from 0 to 0.5 (0.1 interval, figure 4(b)) [17], and the bandgap of (Gd_0.5_Lu_0.5_)AG has been close to that (∼6.40 eV) of a YAG single crystal [27]. The results may also imply that the luminescence property of a (Gd, Lu)AG based phosphor can be finely tuned by varying the Lu content and the onset of optical transmittance of a transparent (Gd, Lu)AG bulk (single crystal or transparent ceramic) would shift towards a shorter wavelength with increasing Lu incorporation.

**Figure 5.**
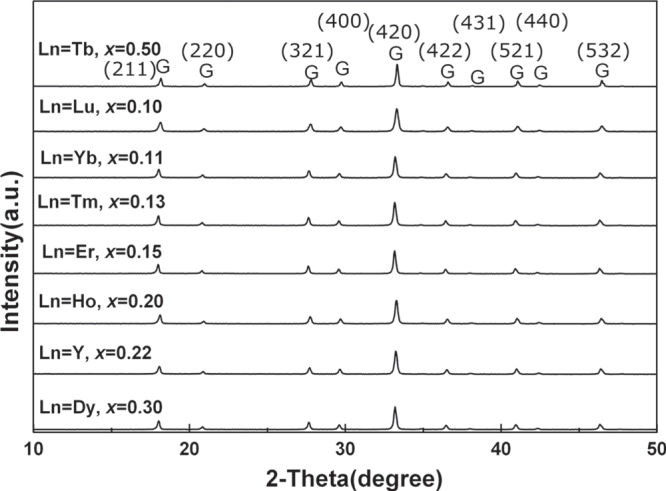
Powder XRD patterns of the phase pure (Gd_1−*x*_Ln_*x*_)AG garnets obtained by doping GAG with the calculated amount (*x* value) of different Ln^3+^. Reproduced with permission from [33], copyright 2013 by Trans Tech Publications.

**Figure 6.**
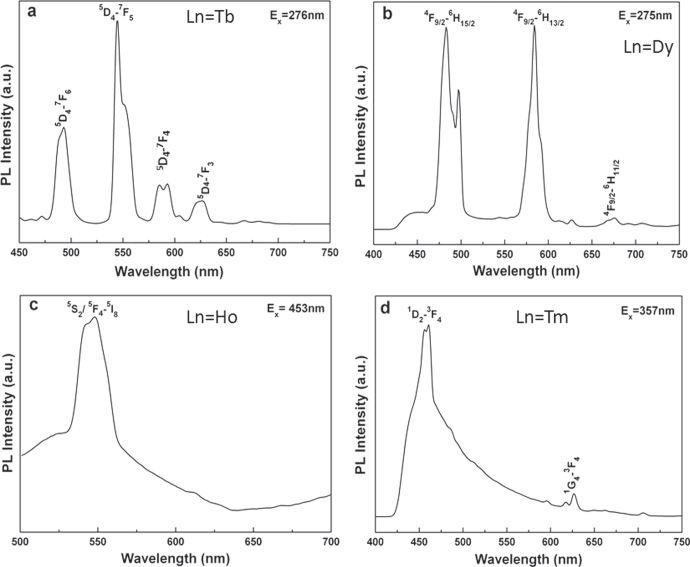
Emission spectra for the (Gd_0.5_Ln_0.5_)AG solid solution, with the excitation wavelength (*E*_*x*_) and the origin of luminescence indicated. Reproduced with permission from [33], copyright 2013 by Trans Tech Publications.

Tb^3+^ is the largest single Ln^3+^ for a stable LnAG to be formed, and thus its size can be taken as a reference for a lattice stabilization study of GAG. The minimum amount of Lu^3+^ (∼17 at%) calculated from the ionic size of Tb^3+^ (0.1040 nm for CN = 8), however, is significantly larger than the ∼10 at% found in practice (figure 3(b)). This indicates that stable garnet solid solutions exist if the average ionic size of (Ln_1_,Ln_2_)^3+^ pair lies in between those of Gd^3+^ (0.1053 nm for CN = 8) and Tb^3+^, in agreement with the fact that TbAG [28–30] and even (Gd_0.9_Lu_0.1_)AG [31, 32] can be further doped with larger Eu^3+^ (0.1066 nm, CN = 8) and Ce^3+^ (0.1143 nm, CN = 8) for luminescence. Taking the average ionic size of (Gd_0.9_Lu_0.1_)^3+^ (~0.1045 nm) as a standard, Li *et al* [33] analyzed the minimum amounts of various small Ln^3+^ that are needed for GAG stabilization, and the *x* value was predicted to be ∼0.5 for Tb^3+^, 0.3 for Dy^3+^ (0.1027 nm), 0.22 for Y^3+^ (0.1019 nm), 0.2 for Ho^3+^ (0.1015 nm), 0.15 for Er^3+^ (0.1004 nm), 0.13 for Tm^3+^ (0.0994 nm), and 0.11 for Yb^3+^ (0.0985 nm). Practical powder synthesis indeed shows that (Gd_1−*x*_Ln_*x*_)AG garnet can be obtained in high phase purity with incorporation of the calculated amount of dopant in each case (figure 5). The results may thus lay a base for flexible materials design by properly combining different types of stabilizers to achieve diverse optical functionalities. The characteristic emission of Ln^3+^ in (Gd_0.5_Ln_0.5_)AG was observed by the authors for Tb (green), Dy (similarly strong blue and yellow), Ho (green), and Tm (blue), despite the high Ln^3+^ concentration (figure 6).

## 3. Down-conversion (DC) phosphors based on GAG

DC phosphors are generally referred to those that absorb high-energy photons and re-emit them at longer wavelengths in the visible range. There are many host lattices for DC luminescence, and the most extensively employed ones may include single-/multi-cation oxides, oxysulfides, phosphates, vanadates, borates, and molybdates/tungstates. Different hosts are used in practice to meet different application needs. Though almost all of the optically active Ln^3+^ can be used as the activator for DC luminescence, the four ions of Ce^3+^, Eu^3+^, Tb^3+^, and Dy^3+^ are the most efficient since their energy gaps between the lowest emission state and ground state are sufficiently wide to avoid significant non-radiative cross relaxations. The emission behaviors of these activators are generally governed by their site symmetry in the host lattice, lattice covalency, lattice defects/distortion, and particle size/shape (surface effects).

### 3.1. Eu^3+^ doping for red luminescence

**Figure 7.**
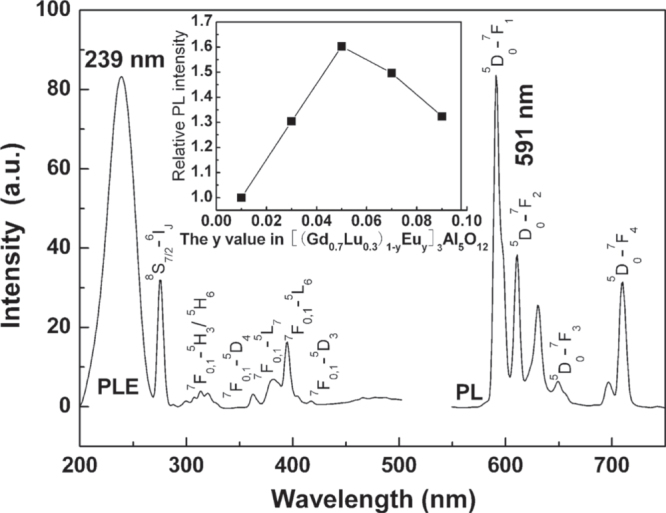
Excitation and emission behaviors of the [(Gd_0.7_Lu_0.3_)_1−*y*_Eu_*y*_]AG red phosphors. Reproduced with permission from [31], copyright 2012 by the National Institute for Materials Science.

Eu^3+^ is well known for its orange red/red emission arising from the ^5^D_0_ → ^7^F_1,2_ electronic transitions. The Eu^3+^ ions doped in LnAG are assumed to replace the Ln^3+^ sites and thus inherit a D_2_ point symmetry, which is only slightly perturbed from the highly symmetric D_2h_ point group [34]. For this, the emission of YAG:Eu and LuAG:Eu is dominated by the parity-law allowed ^5^D_0_ → ^7^F_1_ magnetic dipole transition at ∼590 nm rather than the forced ^5^D_0_ → ^7^F_2_ electric dipole transition at ∼610 nm as observed from the well-known Y_2_O_3_:Eu red phosphor. A [(Gd_1−*x*_Lu_*x*_)_1−*y*_Eu_*y*_]AG solid solution has recently been developed as efficient red phosphor with Lu^3+^ as the lattice stabilizer, and the effects of various factors on optical properties were thoroughly investigated [31]. Taking [(Gd_0.7_Lu_0.3_)_1−*y*_Eu_*y*_]AG for example, the material was shown to be efficiently excitable with the charge transfer band (CTB) at ∼239 nm to produce a sharp orange–red emission at 591 nm (figure 7), with CIE chromaticity coordinates of (0.62, 0.38) and a full width at half maximum of only ∼6 nm for the emission peak. The optimal Eu^3+^ content was experimentally determined to be ∼5 at% (*y* = 0.05), and the quenching mainly resulted from exchange interactions, possibly via phonon assisted three Eu^3+^ ion nonresonant interactions. Greatly improved emission intensity and quantum yield, shortened fluorescence lifetime, and increased asymmetry factor of luminescence (the *I*_591_/*I*_610_ intensity ratio) were observed along with increasing synthesis temperature up to 1500 °C [31], primarily owing to lattice perfection, defect elimination, and particle growth. The [(Gd_0.7_Lu_0.3_)_0.95_Eu_0.05_]AG phosphor synthesized at 1500 °C has internal/external quantum efficiencies (%) of 83.2/56.1, an asymmetry factor of ∼2.85, and a fluorescence lifetime of ∼4.1 ms for the 591 nm emission [31]. The lifetime is close to that (4.66 ms) reported for YAG:Eu [35] but is significantly longer than those (generally 0.5–2.5 ms) of the well-known red phosphors of Y_2_O_3_:Eu [36, 37], (Gd_1−*x*_Ln_*x*_)_2_O_3_:Eu (Ln = Y, Lu) [22, 38], and La_2_O_2_S:Eu [39], since the Eu^3+^ activator takes the highly symmetric D_2_ lattice site in garnet. Increasing Lu incorporation up to *x* = 0.5 would lower excitation/emission and also blue-shift the CTB due to gradually decreased covalency of the host lattice (*χ* = 1.27 for Lu^3+^), for which a minimized Lu content was recommended as long as the garnet lattice can be effectively stabilized [31]. Similar phenomena were observed in the development of (Gd_1−*x*_Ln_*x*_)_2_O_3_:Eu red phosphors (Ln = Y, Lu) [38]. Compared with YAG:Eu, the GAG-based phosphor (figure 7) has an additional excitation band arising from the ^8^S_7/2_ → ^6^I_*J*_ Gd^3+^transition at ∼275 nm (significantly stronger than the strongest ^7^F_0,1_ → ^5^L_6_ intra-4f^6^ transition of Eu^3+^ at ∼395 nm), suggesting substantial energy migration from Gd^3+^ to Eu^3+^. The advantage of GAG over YAG as a host lattice was demonstrated in another study by the authors [40]. For example, the internal quantum yield (∼76%) of [(Gd_0.9_Lu_0.1_)_0.95_Eu_0.05_]AG is appreciably higher than that (∼71%) of (Y_0.95_Eu_0.05_)AG at the same temperature of phosphor synthesis. This is primarily owing to the higher lattice covalency of the former, which allows improved excitation absorption and higher probability of electronic transitions. Though [(Gd_1−*x*_Lu_*x*_)_0.95_Eu_0.05_]AG has had a sufficiently high theoretical density and emission intensity, its fluorescence lifetime is too long for scintillation. Shortening the lifetime to below ∼1.0 ms via codoping (such as Pr^3+^) seems necessary for it to compete with the commercialized (Y,Gd)_2_O_3_:Eu scintillator [41].

**Figure 8.**
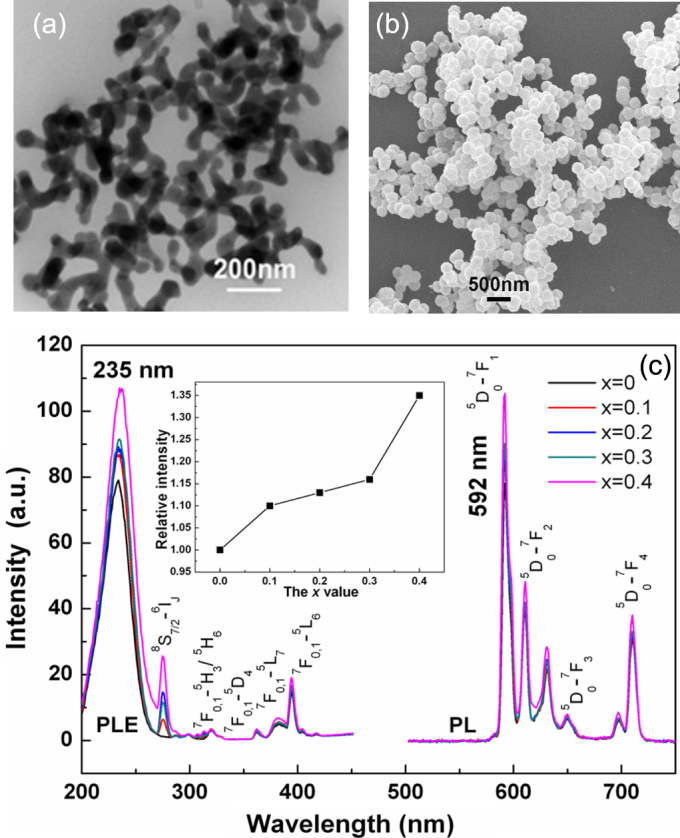
Typical TEM (a) and SEM (b) morphologies of the [(Lu_1−*x*_Gd*_x_*)_0.95_Eu_0.05_]AG red phosphor particles obtained via carbonate coprecipitation and urea-based homogeneous precipitation, respectively. Part (c) shows that the emission intensity of the phosphor spheres improves with increasing Gd content. Parts (a) and (b) reproduced from [31], copyright 2012 by the National Institute for Materials Science.

The carbonate coprecipitation technique, with ammonium bicarbonate as the precipitant, has been able to produce low-aggregation garnet powders, but the primary particles are not separable from each other and are not in a spherical shape (figure 8(a)). Current advances in high-resolution displays not only need finer phosphor particles to improve the resolution by decreasing pixel size but also prefer a spherical particle shape to build a uniform luminescence screen and to improve the brightness of luminescence by minimizing the light scattering on particle surfaces. Urea-based homogeneous precipitation (UBHP) frequently finds success in synthesizing well-dispersed spherical particles of uniform size (monosheres) for single-/multi-cation oxides of the lanthanides [19, 42–48], but failed for YAG in most of the previous studies [49, 50] owing to substantially different solution chemistries of the constituent Y^3+^ and Al^3+^ ions. YAG:Ce phosphor microspheres are thus alternatively made via crystallizing the glassy microbeads quenched from melt droplets produced with laser heating [51]. Xu *et al* [52] identified that, with nitrate as the rare-earth source in UBHP, the aluminum source plays an essential role in the formation of precursor microspheres for YAG:Nd. They found that ammonium aluminum sulfate (alum) is indispensable and the optimal alum/Al(NO_3_)_3_ molar ratio is 1/1. Mechanistic study further revealed that microspheres of the Al component are formed at the early stage of precipitation, followed by Y^3+^ precipitation as basic carbonate. Annealing the sulfate-containing precursor at 1100 °C produced YAG:Nd microspheres that can be densified to a translucent state via vacuum sintering of the dry-compacted green body at 1650 °C for 3.5 h. Such a strategy proved similarly successful for [(Lu_1−*x*_Gd_*x*_)_0.95_Eu_0.05_]AG red phosphor microspheres (*x* ≤ 0.4, figure 8(b)) [53], though Gd^3+^ and Lu^3+^ are different from Y^3+^ in solution chemistry owing to lanthanide contraction. Again, the best results were obtained with alum/Al(NO_3_)_3_ = 1:1 molar ratio. When Al(NO_3_)_3_ is the sole Al source, only a gelatinous precursor that would aggregate into a glasslike hard mass upon drying was produced, implying that the sulfate anions from alum have significantly modified the solution chemistry of cations, particularly that of the significantly smaller Al^3+^, and have taken part in precipitation. When the alum/Al(NO_3_)_3_ ratio is over 1, the primary spheres tend to glue together to form porous precipitates as observed for YAG [50], suggesting that superfluous SO_4_^2−^ may serve as a flocculant. The diameter of [(Lu_1−*x*_Gd_*x*_)_0.95_Eu_0.05_]AG microspheres can be finely tuned from ∼500 to 150 nm by increasing the urea/(Al+Ln) molar ratio from 20 to 100, showing the flexibility of the UBHP technique. A photoluminescence study [53] found successively stronger ^5^D_0_ → ^7^F_1_ emission (591 nm) with increasing *x* (the Gd content), owing to increased lattice covalency (figure 8(c)), and gradually weaker emission at a decreasing particle size owing to surface effects [47, 54].

### 3.2. Ce^3+^ doping for yellow luminescence

**Figure 9.**
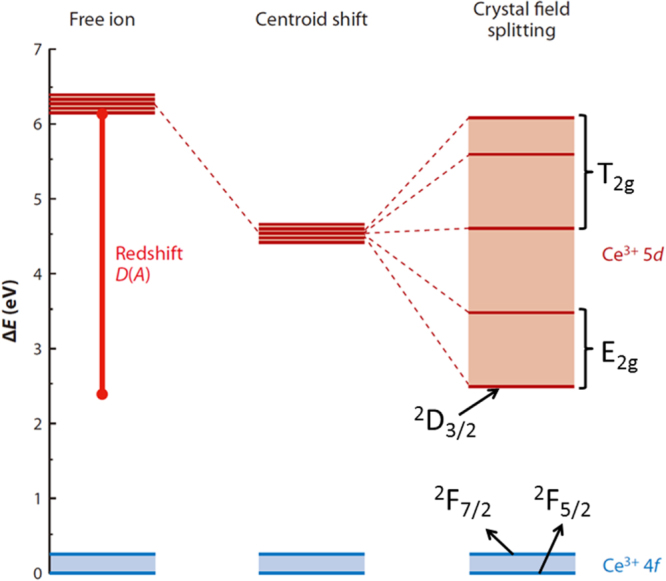
A schematic energy diagram showing the effects of host lattice *A* on centroid shift and crystal field splitting of the Ce^3+^ 5d energy level. Such effects shorten the energy difference between the 5d excited state and 4f ground state, known as red-shift D(*A*). Reproduced with permission from [55], copyright 2013 by Annual Reviews Inc.

**Figure 10.**
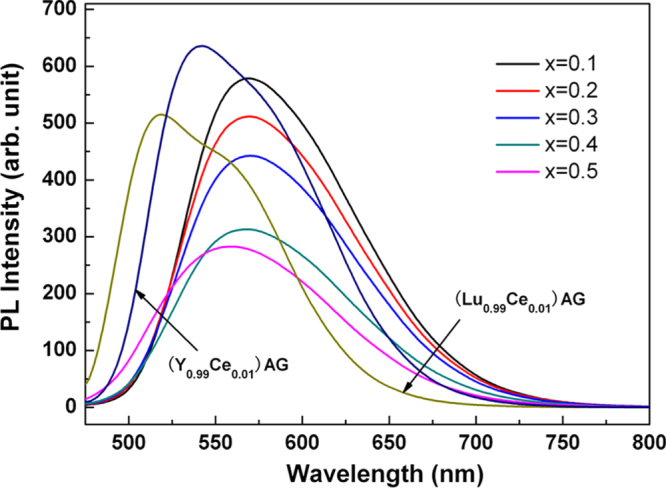
Emission spectra for the [(Gd_1−*x*_Lu_*x*_)_0.99_Ce_0.01_]AG, (Lu_0.99_Ce_0.01_)AG, and (Y_0.99_Ce_0.01_)AG yellow phosphors. Reproduced with permission from [32], copyright 2013 by the National Institute for Materials Science.

**Figure 11.**
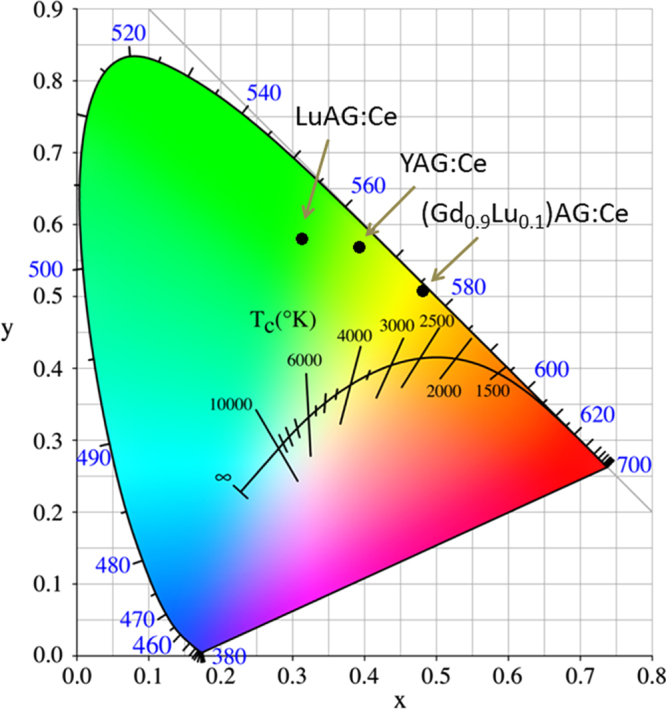
Emission color coordinates for the (Gd, Lu)AG:Ce, YAG:Ce, and LuAG:Ce yellow phosphors.

The yellow emission of Ce^3+^ arises from the 4f^0^5d^1^ → 4f^1^5d^0^ (^2^F_5/2_ and ^2^F_7/2_ ground states) inter-configurational electronic transition. As the exposed 5d electron readily interacts with the surrounding anion ligands, Ce^3+^ emission is strongly influenced by centroid shifting and crystal field splitting of the 5d energy level (figure 9) [55]. YAG:Ce^3+^ has been the most prominent and widely used yellow phosphor in LED lighting, since it can be efficiently excited with (Ga, In)N blue LED chips (∼450 nm) and exhibits a high quantum yield of ∼90% for its ∼540 nm emission [7]. One shortcoming is that, for warm-white lighting, YAG:Ce^3+^ has low color rendering and high correlated color temperature due to its lack of a sufficient red portion in the emission spectrum. To overcome this, partially replacing the Y^3+^ sites with less electronegative La^3+^ or Gd^3+^ [6, 56] and more recently doping YAG with Si_3_N_4_ to form the oxynitride solid solution of Y_2.925_Ce_0.075_Al_5−*x*_Si_*x*_O_12−*x*_N_*x*_ (*x* < 0.4 for phase pure garnet) [57] were shown to be able to push down the lowest crystal-splitting component (^2^D_3/2_) of the 5d^1^ excited level to yield red-shifted emissions. Red-shifting can also be achieved by partially substituting Al^3+^ with Mg^2+^–Si^4+^ pairs on the octahedral and tetrahedral sites, respectively, to enhance lattice covalency [58, 59]. Alternatively, red-shifted Ce^3+^ emission can be directly attained with a more covalent host lattice, such as TbAG and GAG-based garnets. With single crystal films, Zorenko *et al* [29] found that TbAG:Ce exhibits broad band emission peaking at 550 nm under 470 nm excitation [4f^1^(^2^F_5/2_) → 5d^1^(E_2g_) Ce^3+^ transition], with light yields of ∼62–71% depending on the Ce^3+^ content. An efficient Tb^3+^ → Ce^3+^ energy transfer was identified through directly exciting the Tb^3+^ ions in the host lattice at 325 nm. Chiang *et al* [23] found that the emission wavelength of Ga^3+^-stabilized (Gd_0.97_Ce_0.03_)_3_(Al_1−*x*_Ga_*x*_)_5_O_12_ yellow phosphors gradually shortens from ∼565 to 552 nm (*λ*_ex_ = 470 nm) with increasing Ga^3+^ substitution from *x* = 0.1 to 0.3, yet substantially longer than the ∼540 nm emission of YAG:Ce, and the shortening was ascribed to the higher electronegativity of Ga^3+^ (*χ* = 1.81) than Al^3+^ (*χ* = 1.61). Li *et al* [32] studied in detail the synthesis and optical properties of [(Gd_1−*x*_Lu_*x*_)_1−*y*_Ce_*y*_]AG yellow phosphors. It was found that 1 at% (*y* = 0.01) of much larger Ce^3+^ (0.1143 nm) can be doped into the garnet lattice in the presence of 10 at% of Lu (*x* = 0.1) and more Ce^3+^ needs more Lu^3+^. The optimal Ce^3+^ concentration was experimentally determined to be ∼1 at%, and luminescence quenching mainly resulted from exchange interactions. Intensity ratio (*I*_*b*_/*I*_*a*_) of the 460 nm [4f^1^(^2^F_5/2_) → 5d^1^(E_2g_)] to 340 nm [4f^1^(^2^F_5/2_) → 5d^1^(T_2g_)] excitations was observed to significantly increase from ∼4.8 at *y* = 0.01 to ∼9.0 at *y* = 0.02 and then to ∼12 at *y* = 0.03, due to successively stronger non-radiative absorptions. Energy transfer from Gd^3+^ to Ce^3+^ was identified from the appearance of ^8^S_7/2_ → ^6^I_*J*_ Gd^3+^ transition at ∼275 nm. Figure 10 compares the emission spectra of [(Gd_1−*x*_Lu_*x*_)_0.99_Ce_0.01_]AG (λ_ex_ = 455 nm), (Y_0.99_Ce_0.01_)AG (*λ*_ex_ = 454 nm), and (Lu_0.99_Ce_0.01_)AG (*λ*_ex_ = 448 nm), from which it is seen that the emission covers the broad range of ∼475–750 nm in each case and the peak wavelength of [(Gd_1−*x*_Lu_*x*_)_0.99_Ce_0.01_]AG red-shifts relative to those of YAG:Ce and LuAG:Ce even at the high Lu content of 50 at% (*x* = 0.5). Increasing Lu incorporation steadily shortens the emission wavelength due to decreased lattice covalency by the high electronegativity of Lu^3+^ (*χ* = 1.27) and monotonically lowers the emission intensity possibly owing to lattice distortion and defect introduction. The best luminescent [(Gd_0.9_Lu_0.1_)_0.99_Ce_0.01_]AG has an integrated emission intensity ∼97% of (Y_0.99_Ce_0.01_)AG and ∼128% of (Lu_0.99_Ce_0.01_)AG at the same temperature of powder synthesis. The excellent emission, high theoretical density, and relatively low cost of [(Gd_0.9_Lu_0.1_)_0.99_Ce_0.01_]AG may allow it to compete with YAG:Ce and particularly LuAG:Ce for scintillation applications. CIE chromaticity coordinates (figure 11) of the three phosphors are around (0.48, 0.51), (0.39, 0.57), and (0.31, 0.58), corresponding to color temperatures of ∼3044, 4612 and 6010 K, respectively. The chromaticity data again confirm that the GAG-based yellow phosphor has a stronger red component in its emission and is more desirable for warm-white LED lighting.

### 3.3. Tb^3+^ doping for green luminescence

When a Tb^3+^ activator is excited with light of sufficient energy, such as UV light, its 4f^8^ electrons would be raised to the higher 4f^7^5d^1^ level and then fed to the ^5^D_3_,_4_ excited states, from which fluorescence is produced by transitions to the ^7^F_*J*_ (*J* = 1–6) ground states. Though the excited 5d electron is exposed, Tb^3+^ transitions involve only a redistribution of electrons within the inner 4f sub-shell [60], and thus similar emissions are usually observed from different types of host lattices, with the ^5^D_4_ → ^7^F_5_ green emission at ∼545 nm being dominant.

**Figure 12.**
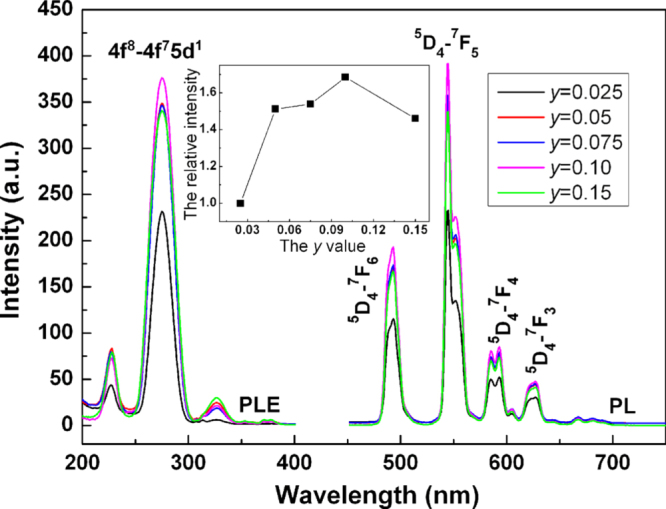
Excitation (*λ*_em_ = 545 nm) and emission (*λ*_ex_ = 276 nm) spectra for the [(Gd_0.8_Lu_0.2_)_1−*y*_Tb_*y*_]AG green phosphors.

YAG:Tb green phosphors are widely studied for applications in cathode ray tubes and flat panel displays such as FED and electroluminescent display, since it is thermally stable and resists saturation under high-current excitations [61, 62]. The GAG-based green phosphor of (Gd, Lu)AG:Tb could be an alternative choice for these purposes and improved performance might also be expected from the possible Gd^3+^ → Tb^3+^ energy transfer. The effects of Tb^3+^ content on photoluminescence of [(Gd_0.8_Lu_0.2_)_1−*x*_Tb_*x*_]AG were studied in figure 12, from which the quenching concentration was determined to be ∼10 at% (*x* = 0.1), almost identical to that of YAG:Tb [63], and luminescence quenching was suggested to occur via exchange interactions [53]. The excitation spectrum consists of three 4f^8^ → 4f^7^5d^1^ transition bands at ∼227 nm (*E*^1–2^_3_ level, spin allowed), 276 nm (*E*^1–3^_2_ level, spin allowed), and 323 nm (*E*_1_ level, spin forbidden), with the 276 nm one being dominant as widely observed [29]. It should be noted that the ^8^S_7/2_ → ^6^I_*J*_ Gd^3+^ transition (∼275 nm) well overlaps the 276 nm excitation, suggesting the likelihood of Gd^3+^ → Tb^3+^ energy transfer, since the ^6^I_*J*_ state of Gd^3+^ lies higher than the ^5^D_3,4_ emission states of Tb^3+^ in the energy diagram of excited states for Ln^3+^ [64–66]. The emission spectrum obtained under 276 nm excitation has four groups of bands at ∼490 (blue), 545 (green, the strongest), 589 (yellow), and 623 nm (red), corresponding to the ^5^D_4_ → ^7^F_6,5,4,3_ transitions, respectively. Emission from the higher ^5^D_3_ excited level, usually in the 450–490 nm region, is hardly observable, which can be explained by cross-relaxation via resonance between the excited and ground states of two Tb^3+^ ions, that is, populating the ^5^D_4_ level by quenching the ^5^D_3_ level via Tb^3+^ (^5^D_3_) + Tb^3+^ (^7^F_0_) → Tb^3+^ (^5^D_4_) + Tb^3+^ (^7^F_6_) [67]. Emission from the ^5^D_3_ state was experimentally found for YAG doped with 1 at% [61] but not with 5 at% of Tb^3+^ [62], and 1 at% is generally accepted as the up-limit for the ^5^D_3_ emission to appear in many hosts. It is noteworthy that the charge transfer state (CTS) of the host lattice also determines the occurrence of ^5^D_3_ emission [68]. In La_2_O_2_S:Tb^3+^, for example, it is completely quenched even at very low Tb^3+^ concentrations, not owing to cross relaxation but by thermal excitation of the ^5^D_3_ electrons into CTS since the two states have similar energies [68]. Comparative studies showed that the [(Gd_1−*x*_Lu_*x*_)_0.9_Tb_0.1_]AG phosphors with *x* = 0.1 and 0.2 have emission intensities close to (Y_0.9_Tb_0.1_)AG and (Lu_0.9_Tb_0.1_)AG, though the latter two have better crystallinity owing to their ease of crystallization, and have fluorescence lifetimes of ∼3.31 ms (3.18 ms for YAG:Tb) and CIE color coordinates of (0.35, 0.57) [53]. Electron-beam excited luminescence of (Gd,Lu)AG:Tb^3+^ is yet needed to study for the aforesaid applications.

### 3.4. Dy^3+^ doping for white luminescence

**Figure 13.**
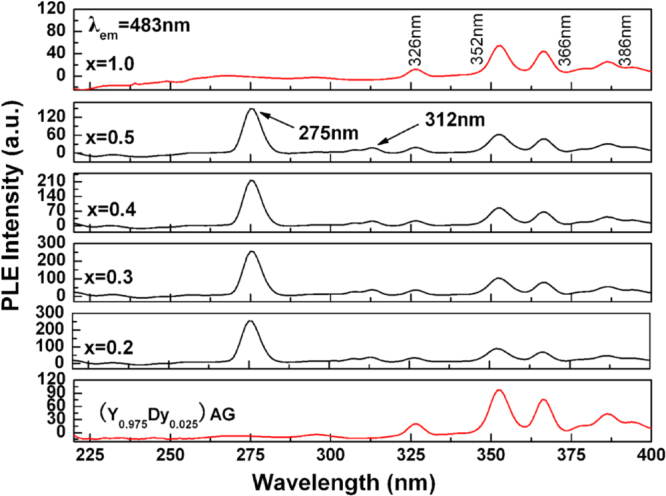
Excitation spectra for the [(Gd_1−*x*_Lu_*x*_)_0.975_Dy_0.025_]AG, (Lu_0.975_Dy_0.025_)AG, and (Y_0.975_Dy_0.025_)AG white phosphors (*λ*_em_ = 483 nm). Reproduced with permission from [74], copyright 2013 by the Royal Society of Chemistry.

**Figure 14.**
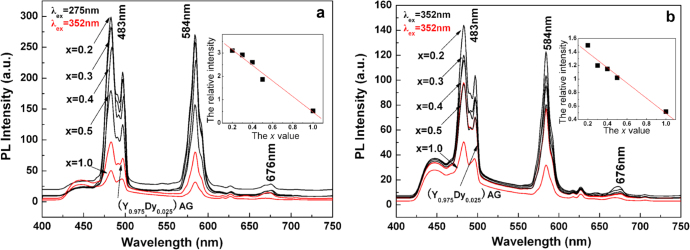
Emission spectra for the [(Gd_1−*x*_Lu_*x*_)_0.975_Dy_0.025_]AG, (Lu_0.975_Dy_0.025_)AG, and (Y_0.975_Dy_0.025_)AG white phosphors, taken under excitations with the ^8^S_7/2_ → ^6^I_*J*_ Gd^3+^ transition at 275 nm (part (a)) and the intra-4f^9^ Dy^3+^ transition at 352 nm (part (b)). Reproduced with permission from [74], copyright 2013 by the Royal Society of Chemistry.

The primary interest in using Dy^3+^ as an activator is that it simultaneously emits blue (∼483 nm, ^4^F_9/2_→^6^H_15/2_ transition) and yellow (∼584 nm, ^4^F_9/2_ → ^6^H_13/2_ transition) lights, which are needed to develop white light in LEDs and optical display systems [69]. Dy^3+^-containing compounds are also used as thermographic phosphors to measure surface temperature by applying a thin coating of the phosphor to the substrate [70]. The luminescence behavior of Dy^3+^ is governed by parity law, and in the cubic lattice of Ln_2_O_3_ sesquioxide the emission spectrum is dominated by the yellow band at ∼584 nm [71, 72]. This is because the blue (parity allowed) and yellow (parity forbidden) emissions come from the Dy^3+^ ions taking symmetric and non-symmetric (or low symmetric) lattice sites, respectively, while in this type of oxide the centrosymmetric S_6_ site has a much lower occupancy (25%) than the non-centrosymmetric C_2_ site (75%) [73]. Relative intensity of the blue emission can be improved in YAG lattice owing to higher site symmetry, but the overall emission intensity is rather limited since within the 4f^9^ configuration of Dy^3+^ the excited electrons have high probabilities of non-radiative cross relaxation owing to the relatively limited energy gap between the excited and ground states and also the abundant energy multiplets for both the states [64, 66]. (Gd,Lu)AG was recently demonstrated to be significantly superior to YAG as the host for Dy^3+^ emission [74]. With the more covalent lattice and particularly via an efficient Gd^3+^ → Dy^3+^ energy transfer, greatly enhanced blue and yellow emissions were simultaneously attained. The optimal Dy^3+^ concentration was found to be ∼2.5 at%, close to the ∼2.0 at% reported for YAG [69], above which luminescence quenching occurs via dipole-dipole interactions. The excitation behaviors of [(Gd_1-*x*_Lu_*x*_)_0.975_Dy_0.025_]AG, (Y_0.975_Dy_0.025_)AG, and (Lu_0.975_Dy_0.025_)AG (*x* = 1.0) are compared in figure 13, where the intra-4f^9^ excitations of Dy^3+^ are similarly found at ∼326, 352, 366, and 386 nm for the ^6^H_15/2_ to ^6^P_3/2_, ^4^I_11/2_+^4^M_15/2_+^6^P_7/2_, ^4^P_3/2_+^6^P_3/2_,_5/2_, and ^4^I_13/2_+^4^F_7/2_+^4^K_17/2_+^4^M_19/2,21/2_ transitions, respectively. It is also seen that the main excitation at 352 nm is generally stronger for [(Gd_1−*x*_Lu_*x*_)_0.975_Dy_0.025_]AG than (Y_0.975_Dy_0.025_)AG and particularly (Lu_0.975_Dy_0.025_)AG owing to the lower electronegativity of the (Gd_1−*x*_Lu_*x*_)^3+^ pair. A significant difference is that [(Gd_1-*x*_Lu_*x*_)_0.975_Dy_0.025_]AG has an additional excitation band at 275 nm, being the strongest in the whole excitation spectrum, that corresponds to the ^8^S_7/2_ → ^6^I_*J*_ Gd^3+^ transition, indicating the happening of efficient Gd^3+^ → Dy^3+^ energy transfer. The ^8^S_7/2_→ ^6^P_*J*_ Gd^3+^ transition appears at ∼312 nm. Figure 14 compares luminescence spectra of the three types of phosphors, from which it is seen that neither the Lu content nor excitation wavelength (275 or 352 nm) brings about appreciable change to the peak position. Emission intensity of the Gd-containing phosphor under 275 nm excitation is roughly two times that under 352 nm excitation, implying that the energy transfer is of high efficiency. Exciting the most luminescent [(Gd_0.8_Lu_0.2_)_0.975_Dy_0.025_]AG phosphor under 275 nm produced an emission intensity roughly six and three times those of (Lu_0.975_Dy_0.025_)AG and (Y_0.975_Dy_0.025_)AG under 352 nm excitation, respectively (figure 14(a)). Even under identical excitation at 352 nm, the emission intensity of [(Gd_0.8_Lu_0.2_)_0.975_Dy_0.025_]AG is about 3.1 and 1.5 times those of (Lu_0.975_Dy_0.025_)AG and (Y_0.975_Dy_0.025_)AG, respectively (figure 14(b)). Furthermore, the [(Gd_1−*x*_Lu_*x*_)_0.975_Dy_0.025_]AG phosphor has color coordinates of (0.33, 0.35), very close to the ideal white point of (0.33, 0.33) in the CIE chromaticity diagram, with a color temperature of ∼5609 K [74].

### 3.5. Eu^3+^/Tb^3+^ codoping for color tunable luminescence

**Figure 15.**
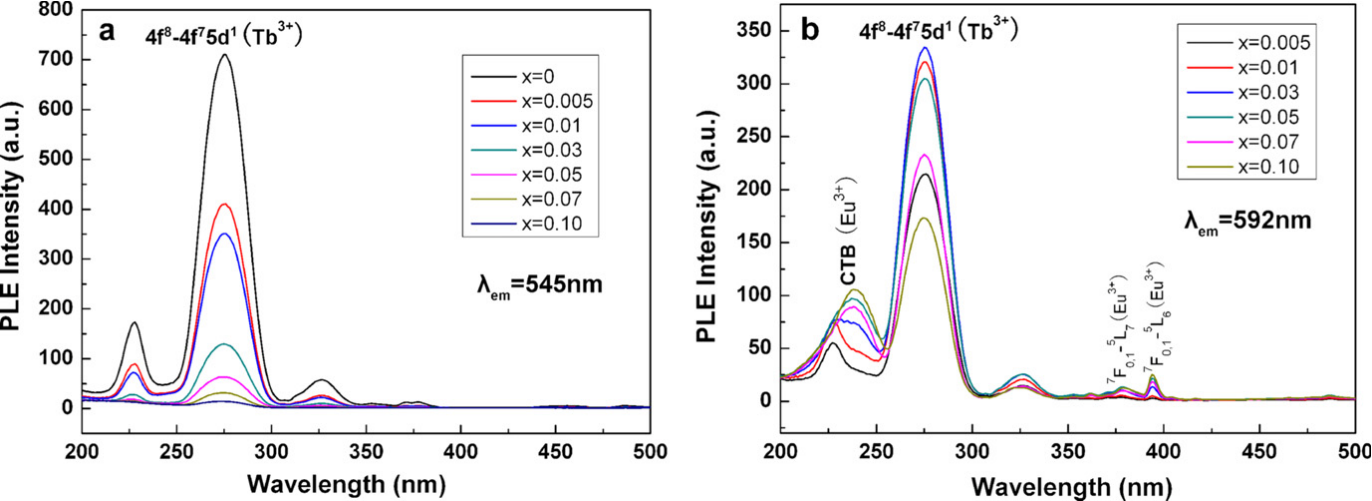
Excitation spectra for the [(Gd_0.8_Lu_0.2_)_0.9−*x*_Tb_0.1_Eu_*x*_]AG phosphors, taken by monitoring the green Tb^3+^ emission at 545 nm (a) and the red Eu^3+^ emission at 592 nm (b).

**Figure 16.**
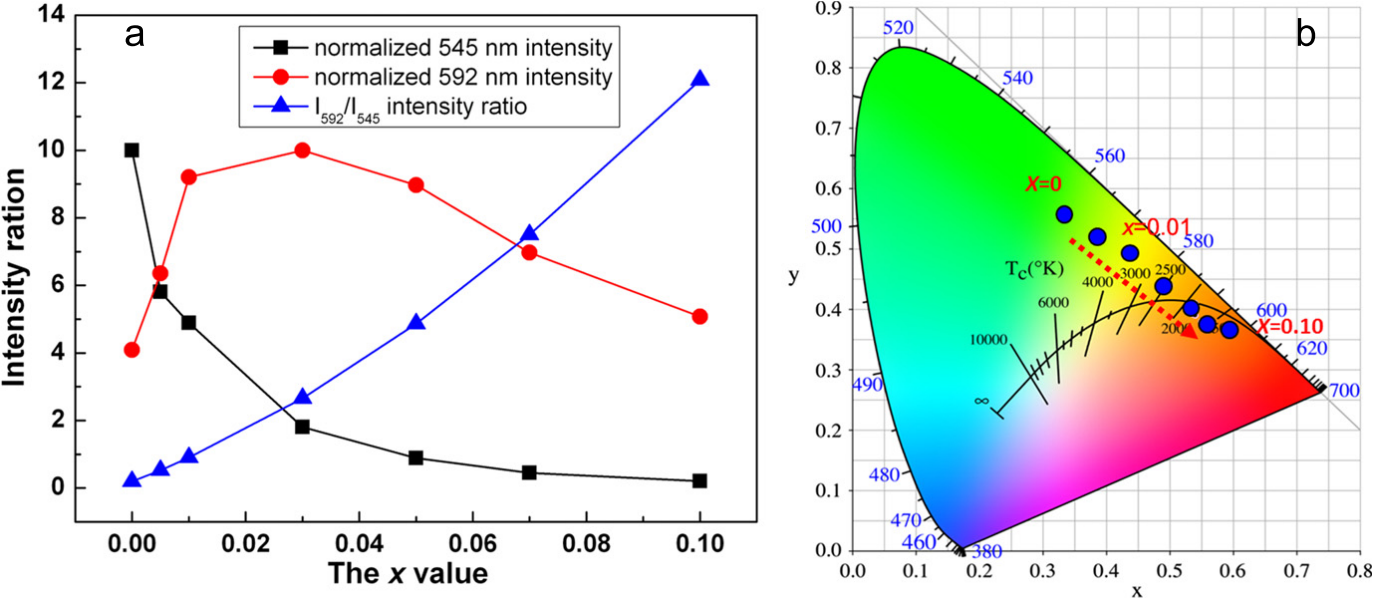
Relative intensities (a) of the 545 nm Tb^3+^ and 592 nm Eu^3+^ emissions and color coordinates (b) of the Tb^3+^/Eu^3+^ co-activated [(Gd_0.8_Lu_0.2_)_0.9−*x*_Tb_0.1_Eu_*x*_]AG phosphors.

**Figure 17.**
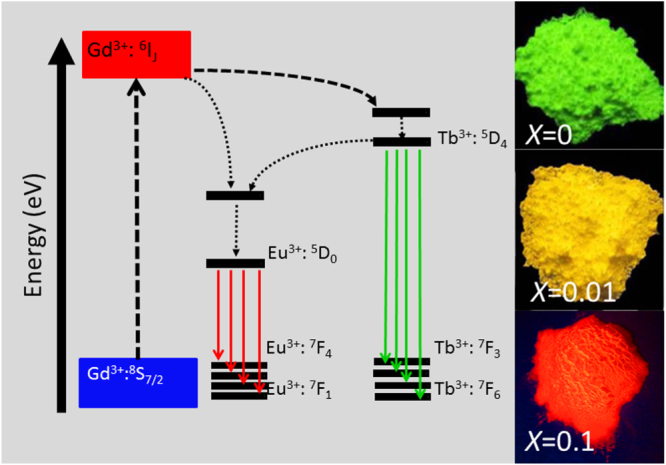
A scheme showing possible pathways of energy transfer (left) in the [(Gd_0.8_Lu_0.2_)_0.9−*x*_Tb_0.1_Eu_*x*_]AG phosphor and digital pictures (right) showing color-tunable emission through the energy transfer (excitation: 275 nm).

Energy transfer between two types of activators is widely utilized in the phosphor field to tune the emission color, to produce a specific color that cannot be attained with one single type of activator, and to enhance the desired emission. The Ce^3+^/Tb^3+^ and Tb^3+^/Eu^3+^ combinations are among the most frequently adopted activator pairs. In the former, the direction of energy transfer largely depends on the 5d^1^ energy level of Ce^3+^, which is as aforesaid readily subjected to centroid shift and crystal field splitting [55]. For example, Ce^3+^ → Tb^3+^ energy transfer is found in CePO_4_:Tb [75] while Tb^3+^ → Ce^3+^ in TbAG:Ce [28, 29]. Dorenbos [76] determined that crystal field splitting of the Ce^3+^ 5d level is affected by coordination geometry, and tends to decrease following the order: octahedral > cubic > dodecahedral > tricapped trigonal prisms and cuboctahedral. Only Tb^3+^ → Eu^3+^ transfer can be observed for the Tb^3+^/Eu^3+^ pair, since the ^5^D_3,4_ excited states of Tb^3+^ lie higher than the ^5^D_0,1_ emission states of Eu^3+^ and both the ions have relatively fixed energy levels for the 4f electrons [64–66]. The Tb^3+^ → Eu^3+^ energy transfer is of high efficiency ( can be ∼90%, for example), because of significant overlapping of the emission spectrum of Tb^3+^ with the excitation spectrum of Eu^3+^ [77, 78]. With such an energy transfer, occurring via electric multipole interactions [78], the emission color of Tb^3+^/Eu^3+^ codoped Y_2_O_3_ can be finely tuned between red and green by varying the atomic ratio of the two activators [78]. Energy transfer and emission control were recently studied for the GAG-based phosphor of [(Gd_0.8_Lu_0.2_)_0.9−*x*_Tb_0.1_Eu_*x*_]AG [53], where the Eu content was varied from *x* = 0 to 0.1. The excitation spectra taken for the Tb^3+^ green emission at ∼545 nm and the Eu^3+^ red emission at ∼592 nm are shown in figure 15. For Tb^3+^ emission (figure 15(a)), only the characteristic excitation bands of Tb^3+^ are resolved, with the inter-configurational 4f^8^ → 4f^7^5d^1^ transition at ∼276 nm being dominant as found for (Gd,Lu)AG:Tb^3+^. Intensity of the excitation significantly decreases with increasing Eu^3+^ addition and finally becomes negligible at *x* = 0.1, primarily owing to Tb^3+^ → Eu^3+^ energy transfer and also concentration quenching at high total contents of the two activators. The excitation spectra taken for Eu^3+^ emission are, however, dominated by Tb^3+^ transitions, and only very weak CTB and intra-4f^6^ transitions originated from Eu^3+^ are found (figure 15(b)). This indicates that, in the codoped system, exciting Tb^3+^ is the only efficient way to produce Eu^3+^ luminescence through energy transfer. Intensity of the 276 nm excitation reaches its maximum at *x* = 0.03, followed by a steady decrease at higher Eu contents owing to concentration quenching. Figure 16(a) analyzes intensities of the 592 nm Eu^3+^ and 545 nm Tb^3+^ emissions (*λ*_ex_ = 276 nm), where the strongest emission is normalized to 10 for both the activators. It is seen that the Tb^3+^ emission is monotonically weakened at a higher Eu content while the Eu^3+^ emission gradually gains intensity up to *x* = 0.03 and then decreases, following the tendency found from the excitation spectra. The *I*_592_/*I*_545_ intensity ratio steadily increases with increasing Eu^3+^ incorporation, which may suggest a persistent energy transfer from Tb^3+^ to Eu^3+^ or the quenching of Eu^3+^ emission is less than that of Tb^3+^. The CIE color coordinates shown in figure 16(b) indicate that the emission can be well tuned from green to orange red via yellow (figure 17). Further analysis indicated that energy transfer may have occurred via electric dipole-quadrupole interactions [53]. It should be noted that the energy process is more complicated for (Gd, Lu)AG than Y_2_O_3_ owing to the presence of optically active Gd^3+^. Since the ^8^S_7/2_ → ^6^I_*J*_ Gd^3+^ transition well overlaps the 4f^8^ → 4f^7^5d^1^ Tb^3+^ transition at ∼276 nm, multichannel energy transfer is highly possible, including Gd^3+^ → Tb^3+^ → Eu^3+^, Tb^3+^ → Eu^3+^ and Gd^3+^ → Eu^3+^ (figure 17), though further studies are needed to clarify the exact routes. The excitation behavior of Eu^3+^ and the significantly lowered Tb^3+^ while improved Eu^3+^ emissions up to *x* = 0.03, however, unambiguously reveal the presence of Tb^3+^ → Eu^3+^ transfer path.

## 4. UC phosphors based on GAG

**Figure 18.**
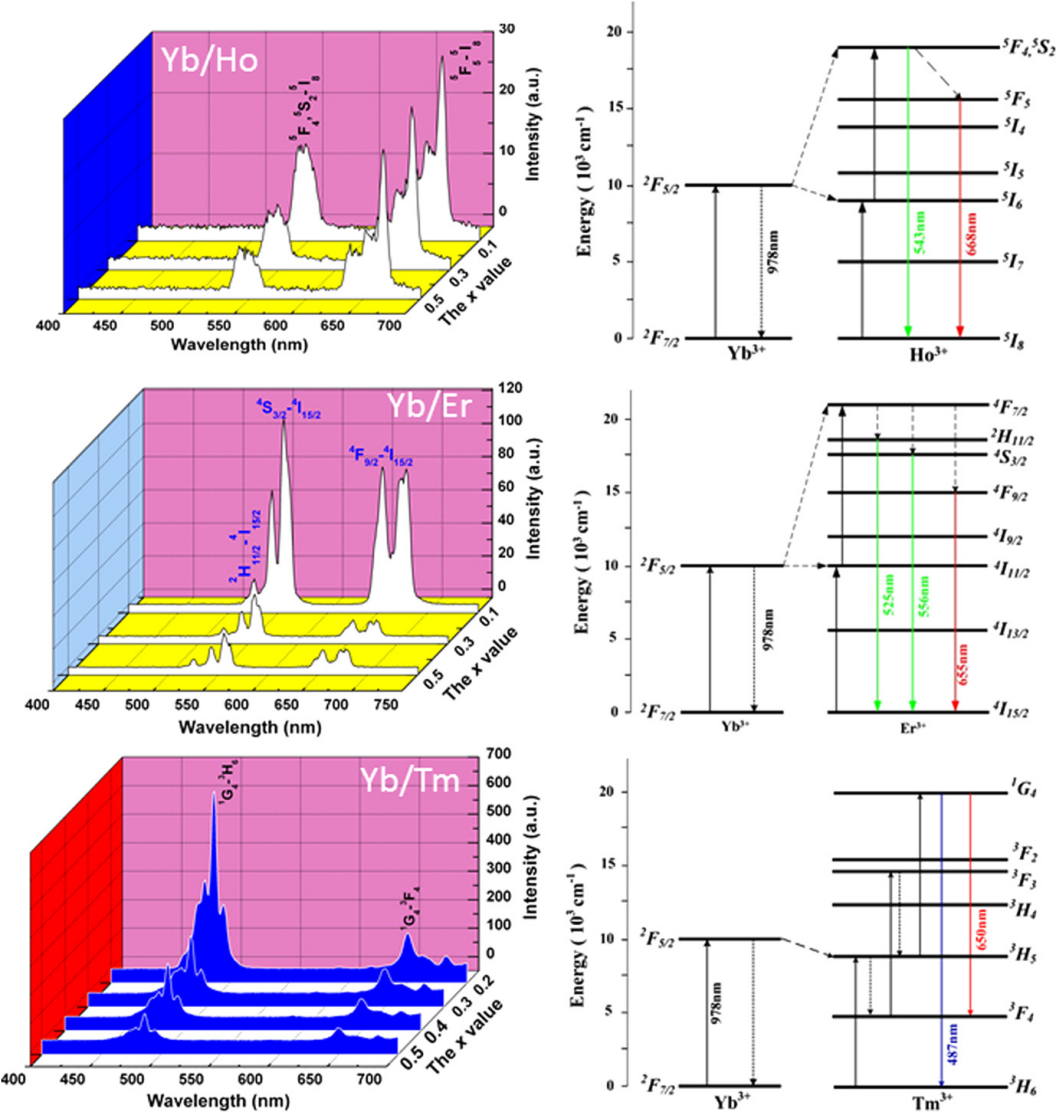
Up-conversion (UC) luminescence spectra of the Yb/Ho, Yb/Er, and Yb/Tm codoped (Gd_1−*x*_Lu_*x*_)AG solid solutions. Mechanisms of the UC processes are presented in the right-hand schemes. Reproduced with permission from [85], copyright 2014 by Trans Tech Publications, and [84], copyright 2014 by Elsevier.

**Figure 19.**
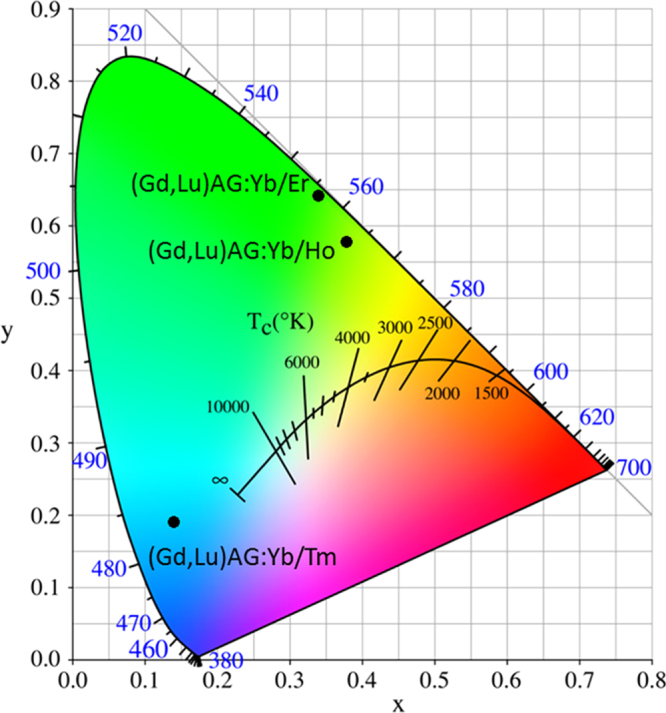
Color coordinates for the UC luminescence of (Gd, Lu)AG:Yb/Ln (Ln = Er, Ho, and Tm).

UC luminescence is an anti-Stokes process in which a longer wavelength radiation, usually near infrared (NIR) or IR light, is converted to a shorter wavelength such as UV or visible light via a two-photon or multi-photon mechanism [79]. The materials are drawing wide interest since they can be used as biological labels for medical diagnosis and therapy, in photovoltaic cells to efficiently harvest solar energy, and also in laser and anti-counterfeit fields [79]. The activators used for UC are those that usually do not exhibit efficient DC luminescence, including Pr^3+^, Sm^3+^, Ho^3+^, Er^3+^, and Tm^3+^. Though when properly doped in a host the above activators themselves have been able to produce UC emission, the efficiency is usually rather limited owing to their unsatisfactory NIR/IR excitations. Yb^3+^ is thus widely employed as a codopant to improve NIR absorption (at ∼980 nm, the ^2^F_7/2_ → ^2^F_5/2_ Yb^3+^ transition) and to sensitize UC emission via nonradiative energy transfer from Yb^3+^ to the activator. The most preferred and widely used hosts for UC luminescence are fluorides owing to their low phonon energy, though other material types, such as Y_2_O_3_ transparent ceramics [80, 81] and Gd_2_O_2_S powder [82], were also explored. YAG was suggested to possess a large ground-state Stark splitting and has a quasi three-level energy structure, which may enable a broad and intense absorption of Yb^3+^ in it [83]. The energy transferred from Yb^3+^ may effectively populate the upper level of Tm^3+^ in YAG [83]. UC performances were recently studied for stabilized GAG with the compositions of [(Gd_1-*x*_Lu_*x*_)_0.948_Yb_0.05_Ln_0.002_]AG (*x* = 0.1–0.5, Ln = Er, Ho, and Tm) [53, 84, 85]. Despite the dilute Yb and Ln concentrations, strong UC luminescence was clearly observed in each case upon laser exciting Yb^3+^ at 978 nm, as shown in figure 18 together with the mechanism of UC. The UC luminescence presents as an intense blue band at ∼487 nm (^1^G_4_ → ^3^H_6_ transition) and a weaker red one at ∼650 nm (^1^G_4_ → ^3^F_4_) for Tm^3+^, as a fairly strong green band at 543 nm (^5^F_4_,^5^S_2_ → ^5^I_8_) and a strong red band at 668 nm (^5^F_5_ → ^5^I_8_) for Ho^3+^, and as three bands at 525 nm (green, ^2^H_11/2_ → ^4^I_15/2_), 556 nm (yellow, ^4^S_3/2_ → ^4^I_15/2_) and 655 nm (red, ^4^F_9/2_ → ^4^I_15/2_) for Er^3+^. CIE chromaticity coordinates of the UC luminescence were found to be around (0.14, 0.19) for Tm^3+^, (0.38, 0.58) for Ho^3+^, and (0.34, 0.64) for Er^3+^, corresponding to blue, greenish yellow, and green colors, respectively (figure 19). Lowered luminescence with increasing Lu incorporation and enhanced emission with increasing temperature of phosphor synthesis were found for the above UC systems. Analyzing the emission intensity against excitation power indicated that the UC luminescence may have occurred via a three-photon process for Tm^3+^ and a two-photon process for both Ho^3+^ and Er^3+^, which are schematically shown in the right part of figure 18 [53, 84, 85].

## 5. Single-crystal and transparent-ceramic scintillators based on GAG

A scintillator is essentially a luminescent material that absorbs high-energy photons and then emits visible light, for which efficient absorption of the excitation source is a fundamental requirement [41, 86]. Since the relation among absorption coefficient (*η*_abs_), theoretical density (*ρ*), and effective atomic number (*Z*_eff_) can be expressed as *η*_abs_ = *ρZ*_eff_^4^ [87], high theoretical density (generally >6 g cm^−3^) and particularly high effective atomic number are thus needed for an excellent scintillator, though other characteristics such as high light yield and fast response (10–100 ns, dominant decay generally <3 *μ*s to avoid signal pile-up with standard shaping electronics) are also essential [86]. Scintillators combined with photodetectors are widely used in various medical imaging technologies, such as x-ray computed tomography, positron emission tomography (PET), and single-photon emission computed tomography (SPECT), and also in high energy and nuclear physics. The most common scintillators up to date are CsI:Tl, CdWO_4_, and Bi_4_Ge_3_O_12_ (BGO) single crystals, but they have their respective shortcomings such as hygroscopicity, poor machinability, insufficient light output, and slow blinking [86]. For these reasons, Ce-doped silicates, such as Gd_2_SiO_5_ (GSO), Lu_2_SiO_5_ (LSO), (Lu_1−*x*_Y_*x*_)_2_SiO_5_ (LYSO), and LaBr_3_ are being developed as alternatives [88–94].

**Figure 20.**
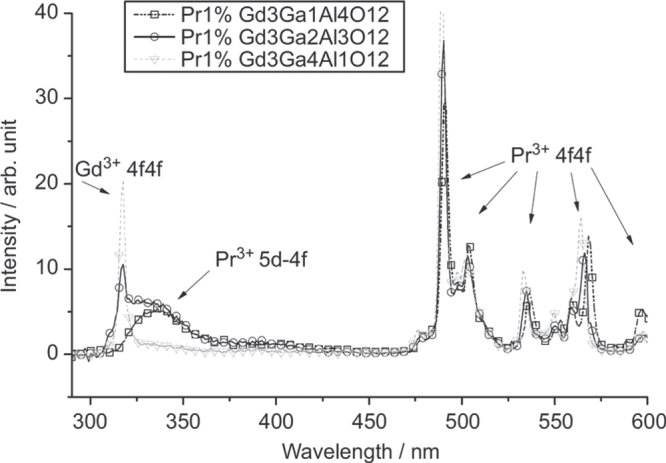
Radioluminescence spectra of the Gd_3_(Al_5−*x*_Ga_*x*_)O_12_:1 at%Pr single crystals under *γ*-ray excitations. Reproduced with permission from [25], copyright 2012 by Elsevier.

**Figure 21.**
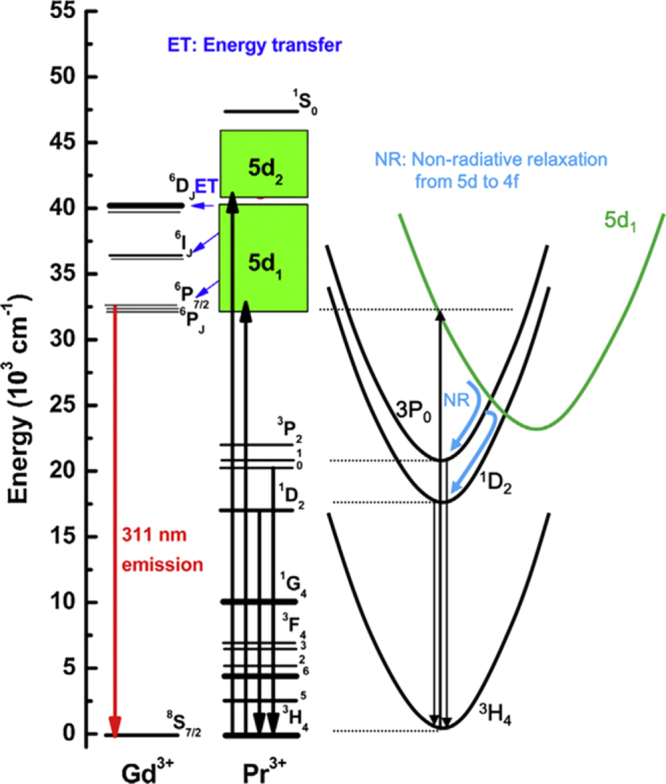
Energy diagram for the Gd^3+^ and Pr^3+^ centers in (Gd_*x*_Lu_3−*x*_)(Ga_3_Al_2_)O_12_ (*x* < 0.2), with the energy transfer channel from Pr^3+^ to Gd^3+^ indicated. The right-hand scheme depicts non-radiative relaxation from the lowest 5d^1^ to low-lying ^3^P_0_ and ^1^D_2_ levels of Pr^3+^. Reproduced with permission from [98], copyright 2013 by Elsevier B V.

**Figure 22.**
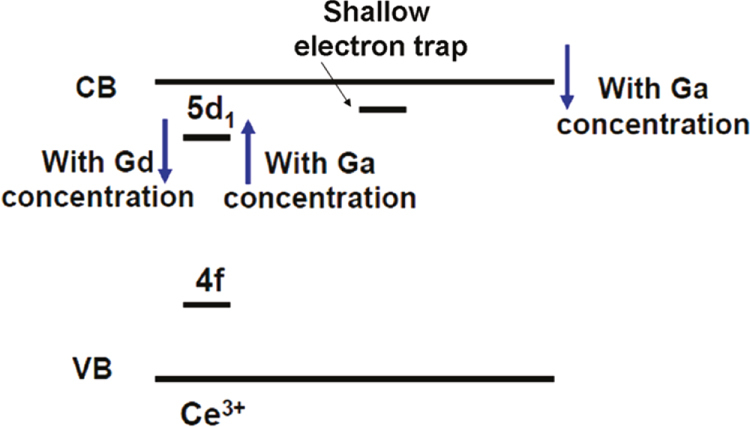
Energy level scheme showing bandgap and 5d^1^ level engineering for the (Gd, Lu)_3_(Ga, Al)_5_O_12_:Ce scintillation crystals. The Ga component helps to lower the conduction band (CB) to bury the shallow traps while the Gd component pushes away the 5d^1^ level of Ce^3+^ from the bottom of CB to avoid Ce^3+^ ionization. Reproduced with permission from [96], copyright 2011 by the American Chemical Society.

GAG-based single crystal scintillators are mostly studied by Kamada *et al* [24, 25, 95–97] through crystal growth by the *μ*-PD and CZ techniques. The CZ-grown Gd_3_(Al_2_Ga_3_)O_12_:1 at%Ce single crystal, where Ga^3+^ is a lattice stabilizer, was reported to have a high light yield of 46 000 photons/MeV for the Ce^3+^ emission (31 000 photons/MeV for LYSO:Ce), an energy resolution of 4.9% at 662 keV, a primary decay time of 88 ns (91%), and a high theoretical density of 6.63 g cm^−3^ [24]. The crystals are thus regarded as promising scintillators for PET, SPECT, and gamma camera. The *μ*-PD grown Gd_3_(Al_5−*x*_Ga_*x*_)O_12_:1 at%Pr single crystals (*x* = 1–5) were found to exhibit the 5d → 4f and intra-4f^2^ transition emissions of Pr^3+^ in the 300–400 and 480–600 nm (dominant) regions, respectively, together with the intra-4f^7^ Gd^3+^ emission at 310 nm (figure 20) [25]. A higher Ga^3+^ content would suppress the 5d → 4f Pr^3+^ emission while enhance the f–f transitions of both Gd^3+^ and Pr^3+^. A low light output of ∼4500 photons/MeV, only about 1/5 of the CZ-grown LuAG:Pr standard, and a relatively long primary decay time of 214 ns (98.8%) were reported for the Gd_3_(Al_2_Ga_3_)O_12_:1 at%Pr single crystal [25]. The poor light output was suggested to be associated with an energy transfer from the 5d state of Pr^3+^ to the 4f state of Gd^3+^ and non-radiative relaxations from the 5d to 4f states of Pr^3+^, with the former being dominant, as schematically shown in figure 21 [98]. Pr^3+^ was thus suggested not to be a proper activator for Gd^3+^-containing scintillators. By simultaneously modifying GAG with Lu^3+^ for Gd^3+^ and Ga^3+^ for Al^3+^, (Gd_2_Lu_1_)(Al_5−*x*_Ga_*x*_)O_12_:Ce single crystals (*x* = 1–5; Ce^3+^ content: 0.2, 1.0, and 3 at%) were grown by the *μ*-PD technique and thoroughly investigated for their luminescence properties [95]. It was shown that decay of the 5d → 4f Ce^3+^ emission at ∼520 nm accelerates with increasing Ga or Ce concentration, and the best composition of (Gd_2_Lu_1_)(Al_2_Ga_3_)O_12_:1 at%Ce has a light yield of ∼22 000 photons/MeV, about 70% of LYSO:Ce (31 000 photons/MeV), a theoretical density of 6.88 g cm^−3^, and a decay time of 76.5 ns (83%) and 282 ns (17%). With the combinatorial approach, Kamada *et al* made comprehensive composition optimization for 0.2 at% Ce^3+^ doped (Gd_3−*x*_Lu_*x*_)(Al_5−*y*_Ga_*y*_)O_12_ [96] and (Gd_3−*x*_Y_*x*_)(Al_5−*y*_Ga_*y*_)O_12_ [97] single crystals. The light output of Ce^3+^ in the best hosts of (Gd_2_Y_1_)(Al_2_Ga_3_)O_12_ and Gd_3_(Al_2_Ga_3_)O_12_ reached ∼42 000–44 000 photons/MeV, being ∼150% of the value of LYSO:Ce and 730% of that of BGO (5700 photons/MeV), with the scintillation decay time dominated by 50–80 ns. The energy resolution at 662 kV was determined to be 8.3% [96], which, though inferior to the 6.7% for high-quality CZ-grown LuAG:Ce, is comparable to the 8.7% measured for LYSO:Ce. Bandgap engineering was pointed out to be crucial in developing high quality scintillators, as also suggested by previous studies. For example, the 5d → 4f luminescence of Ce^3+^ is quenched in the high density garnets of Lu_3_Ga_5_O_12_ (7.4 g cm^−3^) and Gd_3_Ga_5_O_12_ (7.04 g cm^−3^) owing to positioning of the Ce^3+^ 5d states in the conduction band of the host [99], and the performance of LuAG:Ce is strongly degraded by shallow electron traps (Lu_Al_ anti-site defects) via delaying energy transfer to Ce^3+^ and giving rise to quite slow components in the scintillation response [100, 101]. A balanced Ga and Gd admixture may eliminate the trapping effects by burying the shallow traps in the bottom of the conduction band and at the same time avoid ionization of the Ce^3+^ activators by separating the 5d excited level from the conduction band, as schematically shown in figure 22 [96, 97].

**Figure 23.**
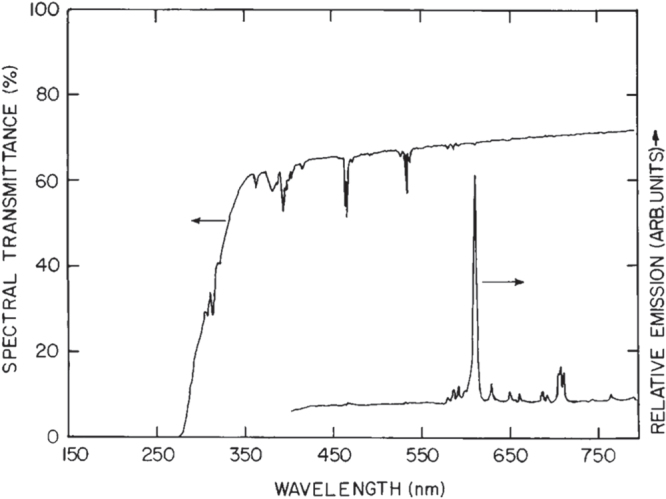
Transmittance and luminescence (excitation: 254 nm UV light) spectra of a 1.5 mm thick (Y_0.67_Gd_0.30_Eu_0.03_)_2_O_3_ ceramic scintillator. Reproduced with permission from [86], copyright 1997 by Annual Reviews Inc.

**Figure 24.**
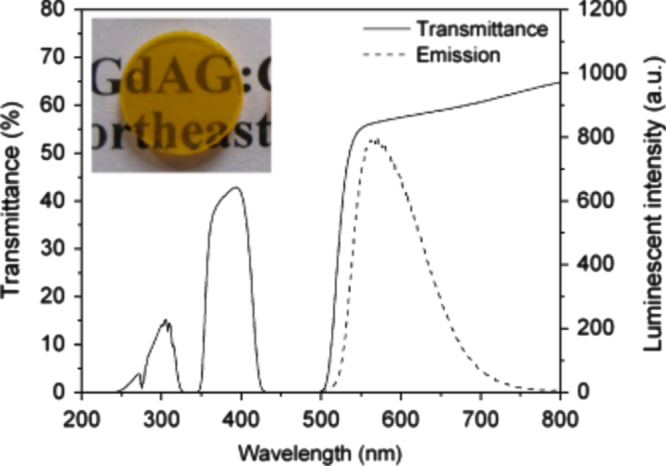
In-line transmittance and luminescence (excitation: 340 nm UV light) spectra of a 1.3 mm thick (Y_1.48_Gd_1.5_Ce_0.02_)AG scintillation ceramic. Reproduced with permission from [104], copyright 2010 by the American Ceramic Society.

**Figure 25.**
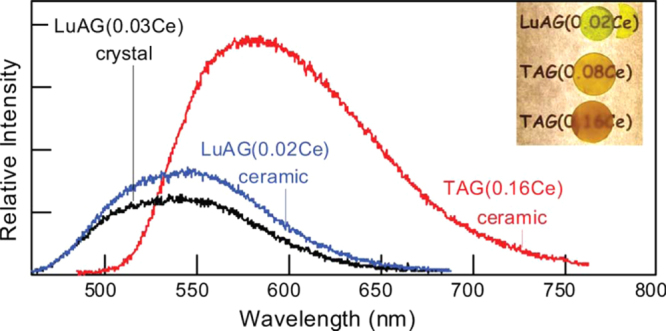
Beta-excited radioluminescence spectra and appearances of the Ce^3+^ doped TAG and LuAG ceramics with scatter mean free path >1 cm. Reproduced with permission from [105], copyright 2009 by IEEE.

**Figure 26.**
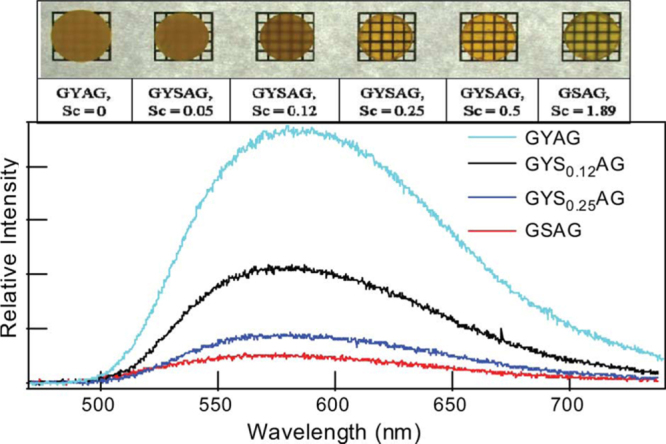
Beta-excited radioluminescence spectra and appearances of the Ce^3+^ doped gadolinium-based garnet ceramics. Reproduced with permission from [105], copyright 2009 by IEEE.

Aside from single crystals, transparent ceramics are under active development for scintillation applications, since the sintering technique generally has advantages in product size and fabrication cost. The (Y,Gd)_2_O_3_:Eu transparent ceramic (inline transmittance ∼73% at the 610 nm emission of Eu^3+^ or ∼90% of the theoretical value, figure 23), fabricated via pressureless sintering, pressureless sintering plus hot isostatic pressing, and vacuum hot pressing, has been the first commercialized polycrystalline scintillator used in medical x-ray detectors [41, 86]. The (Y_0.67_Gd_0.30_Eu_0.03_)_2_O_3_ composition (5.92 g cm^−3^) was reported to have a relative light output 2.5 times higher and ∼30% lower than those of CdWO_3_ and CsI:Tl single crystals, respectively [41]. Li *et al* [102] fabricated transparent (Y_0.3_Gd_0.67_Eu_0.03_)_2_O_3_ ceramic with a higher Gd content, an inline transmittance of ~68% at the 610 nm Eu^3+^ emission and a higher theoretical density of 6.87 g cm^−3^, by vacuum sintering at 1670 °C for 2 h of the oxide powders calcined from coprecipitated carbonate precursors. The main problem associated with Eu^3+^ emission is the long fluorescence decay, which is usually ∼1 ms [41, 86]. Yanagida *et al* [8, 103] made transparent YAG:Ce and (Gd,Y)AG:Ce ceramics for possible scintillation applications (the Gd content and optical transmittance not specified), and the dominant decay time was found to be ∼90 ns under *γ*-ray excitations. Light yield of the latter was reported to be 30% of the former, without specifying the reason, and the energy resolution was found to be ∼8% at 662 keV when coupled with an avalanche photodiode [103]. The stopping power of (Gd, Y)AG:Ce was reported to be five times as high as that of YAG:Ce [103]. Li *et al* [104] studied sintering of (Y_1−*x*_Gd_*x*_)AG:Ce (*x* = 0.01, 0.5, 0.75, and 1; Ce content: 2 at%) transparent ceramics with commercially available nano-sized powders. It was shown that the GAG composition (*x* = 1.0) cracks owing to the stress (volume change) arising from thermal decomposition while all the other compositions can be densified to transparency by vacuum sintering at 1600–1700 °C for 5 h. The (Y_1.48_Gd_1.5_Ce_0.02_)AG ceramic, with an inline transmittance of ∼65% at 800 nm, exhibits Ce^3+^ emission at ∼570 nm under 340 nm UV excitation, which is red-shifted ∼40 nm relative to YAG:Ce (∼530 nm) owing to the high content of less electronegative Gd^3+^ (figure 24). Cherepy *et al* [105] studied radioluminescence properties of translucent (Tb_3−*x*_Ce_*x*_)AG ceramics (figure 25), and much higher light yields (80 000 photons/MeV) than LuAG:Ce (∼30 000 photons/MeV) were found under *β*-ray excitations. The decay, however, is slow owing to energy migration on the Tb^3+^ sites, and the principal decay time reaches <1 *μ*s only at the high Ce^3+^ content of *x* = 0.12. By applying vacuum sintering plus hot isostatic pressing, Cherepy *et al* [105] also fabricated a series of GAG-based scintillation ceramics (Ce^3+^ content: 3 at%), including (Gd_1.5_Y_1.5_)_3_Al_5_O_12_ (GYAG), (Gd_1.5_Y_1.5_)_3_(Al_5−*x*_Sc_*x*_)O_12_ (GYSAG), and Gd_3_(Al_3_Sc_2_)O_12_ (GSAG), using Y^3+^/Sc^3+^ as dopant for lattice stabilization (figure 26). GYAG:Ce was reported to have a very high light yield of ∼100 000 photons/MeV under *β*-ray excitation, due to an efficient energy transfer from Gd^3+^, and have decay time in the 100–200 ns range. Transparency, however, was not achieved for this composition due to the presence of a small amount of GdAlO_3_ secondary phase. In contrast, GSAG and GYSAG only produced the moderate light yields of ∼20 000 photons/MeV, with the primary decay time <200 ns, but formed phase-pure garnet with excellent transparency for the former and acceptable transparency for the latter (*x* ≥ 0.12, transmittance data not shown). In general, the Ce^3+^ emission in Gd-containing scintillators simultaneously has fast primary decay (<200 ns) and high light yield, as compared from table 1 for the typical garnet compounds discussed in this work, showing substantial advantages of the GAG host lattice.

**Table 1.** A summary of the scintillation properties of the promising single-crystal/ceramic scintillators discussed in this work.

Host lattice	Ce content (at%)	Material form	Density (g cm^−3^)	Excitation source	Light yield (photons/MeV)	Resolution at 662 kV (%)	Primary decay (ns)	Reference
LuAG	1.0	Crystal	6.73	*γ*-ray	7500	66.2 (88%)	—	[95]
LuAG	3.0	Crystal	6.73	*γ*-ray	28 000	8.6	—	[105]
				*β*-ray	30 000			
LuAG	2.0	Ceramic	6.73	*γ*-ray	20 000	11.4	—	[105]
				*β*-ray	30 000			
YAG	1.0	Crystal	4.55	*γ*-ray	22 700	—	83.1 (71%)	[97]
YAG	—	Ceramic	4.55	*γ*-ray	30 000	7.3	90	[103, 105]
				*β*-ray	40 000			
TbAG	2-16	Ceramic	—	*γ*-ray	19 500	10.6	4500–600	[105]
				*β*-ray	80 000			
(Gd_1.5_Y_1.5_)Al_5_O_12_	3.0	Ceramic	—	*γ*-ray	16 500	11.2	100–200	[105]
				*β*-ray	>80 000			
Gd_3_(Sc_1.89_Al_3.11_)O_12_	3.0	Ceramic	—	*γ*-ray	7500	10.8	100–200	[105]
				*β*-ray	20 000			
Gd_3_(Al_2_Ga_3_)O_12_	1.0	Crystal	6.63	*γ*-ray	46 000	4.9	88 (91%)	[24]
Gd_3_(Al_2_Ga_3_)O_12_	0.2	Crystal	6.63	*γ*-ray	42 217	8.3	52.8 (73%)	[96]
(Gd_2_Lu_1_)(Al_2_Ga_3_)O_12_	1.0	Crystal	6.88	*γ*-ray	22 000	11.2	76.5 (83%)	[95]
(Gd_2_Y_1_)(Al_2_Ga_3_)O_12_	1.0	Crystal	—	*γ*-ray	44 000	8.2	56.9 (66%)	[97]

## 6. Summary and outlook

Lattice stabilization of GAG (Gd_3_Al_5_O_12_, GAG) and the related development of advanced optical materials are summarized in this article, including down-/up-conversion phosphors, transparent ceramics, and single crystals. It is shown that novel emission features and significantly improved luminescence can be achieved for a number of phosphor systems with the more covalent GAG lattice and the efficient energy transfer from Gd^3+^ to the activator. GAG-based single crystals and transparent ceramics with Ce^3+^ as the activator are shown to have fast scintillation decay and high light yields, and thus hold great potential as scintillators for a wide range of applications. Anti-site defects commonly exist in aluminate garnets [106–108], and their occurrence and energy level have profound influences on various aspects of emission through interacting with the excited electrons. These issues, however, have rarely been tackled for the GAG-based garnets, either when Lu^3+^ or Ga^3+^ is used as lattice stabilizer, and thus need to clarify in future studies for a better control of optical properties. In addition, GAG-based transparent ceramics have been much less developed than their YAG and LuAG counterparts, and need significantly more studies on powder processing and sintering technologies to improve their overall transmittance and other optical performances.
